# An Uncommon Clinical Mystery: Systemic Lupus Erythematosus Masquerading as Aseptic Meningitis in a Male Patient

**DOI:** 10.7759/cureus.70484

**Published:** 2024-09-30

**Authors:** Keshavprakash V, Ashwin Srinivas, Vidya T A, Varadharajan Jayaprakash, Janardanan Kumar

**Affiliations:** 1 Department of General Medicine, Sri Ramaswamy Memorial (SRM) Medical College Hospital and Research Centre, Sri Ramaswamy Memorial (SRM) Institute of Science and Technology, Kattankulathur, Chennai, IND; 2 Department of Nephrology, Sri Ramaswamy Memorial (SRM) Medical College Hospital and Research Centre, Sri Ramaswamy Memorial (SRM) Institute of Science and Technology, Kattankulathur, Chennai, IND

**Keywords:** benign aseptic meningitis, lupus nephritis flare, male sle, neuropsychiatric sle, subnephrotic proteinuria

## Abstract

Systemic lupus erythematosus (SLE) is an intricate autoimmune disease characterized by its impact on various organ systems, presenting with a wide range of clinical manifestations such as hematologic, neuropsychiatric, mucocutaneous, serosal, musculoskeletal, immunologic, cardiac, pleuropulmonary, and renal symptoms. Although its etiology is primarily autoimmune, various triggers, such as pregnancy, certain drugs, and infections, can result in "flares" with frequent relapses. Although more common in females, SLE is not uncommon in males, with a significant proportion experiencing a high disease burden. Over the years, many treatment modalities and approaches in modern medicine have evolved to combat this disease. However, it still poses a challenge to treating physicians due to the intricate elements in its pathogenesis. Further evidence-based studies are necessary to enhance our understanding of the disease. We describe the case of a 53-year-old man who presented with a three-day history of fever and a one-day history of altered sensorium. On evaluation, he was found to have pancytopenia and acute kidney injury. He was worked up for infectious and inflammatory causes. Investigations were strongly in favor of SLE and aseptic meningitis. We started him on pulse steroid therapy, following which he had substantial recovery. After one year, he presented with complaints of frothy urine, when lupus nephritis was diagnosed, and he was started on specific immunosuppressive agents. He has had no further episodes of relapse since then.

## Introduction

Systemic lupus erythematosus (SLE) results from chronic and recurrent activation of the immune system, inflammation, and tissue damage caused by antibody production. It is an autoimmune disease with multisystem involvement [1]. Females have an 8-15 times greater SLE prevalence than males [2]. The prevalence is 14-60 per 100,000 population in India [3]. It is common in young females, but when it occurs in men, it has higher disease activity [1]. Certain environmental factors may contribute to lupus initiation and flare-ups. The production of interferon type I and how it affects the activation and function of the immune system have become a central part of how lupus develops. Hormonal changes also play a major role in pathogenesis. Clinical and immunological criteria are essential for SLE diagnosis. Factors such as a later age of onset, distinct symptoms, and treatment outcomes can make diagnosing and treating SLE in men difficult. Here, we discuss the case of a middle-aged man who was diagnosed with SLE and had a good outcome following appropriate therapy.

## Case presentation

A 53-year-old man with pre-existing conditions of systemic hypertension, type 2 diabetes mellitus, and coronary artery disease presented to the emergency department with complaints of altered mental status with progressive deterioration for the past one day. He had a history of intermittent fever, headaches, and reduced urine output for the past three days. There is no history of visual changes or involuntary movements. He did not have any familial predisposition to autoimmune illness. He reported no recent travel, substance abuse, or other significant medical history.

Upon examination, he was febrile (101 degrees Fahrenheit). Vital parameters were as follows: blood pressure (130/80 mmHg), pulse rate (86 beats/min), respiratory rate (14 cycles/min), SpO_2_ (98% on room air), and capillary blood glucose (180 mg/dL). The Glasgow Coma Scale (GCS) was 13/15 (eye response E3, verbal response V5, motor response M5). Pupils were bilaterally equal and reactive to light; neck rigidity was present. Kernig’s sign was positive, deep tendon reflexes were normal, and plantar was bilateral flexor, with no focal neurological deficits. Other system examinations were unremarkable. Fundoscopic examination revealed mild non-proliferative diabetic retinopathy and no papilledema. Initial laboratory investigations on arrival are summarized in Table 1. 

**Table 1:** Laboratory investigations. WBC: white blood cells; ESR: erythrocyte sedimentation rate; RBC: red blood cells; UPCR: urine protein-creatinine ratio.

Test	Result	Reference value
Hemoglobin	8.5 g/dL	13-17 g/dL
WBC	2,090/cu.mm	4,000-11,000/cu.mm
Platelets	70,000/cu.mm	150,000-450,000/cu.mm
ESR	74 mm	10-12 mm/h
Urea	176 mg/dL	17-43 mg/dL
Creatinine	3.0 mg/dL	0.7-1.3 mg/dL
Urinalysis		
Albumin	++	
RBC	6-7/high power field	
UPCR	1.73	<0.2

A lumbar puncture was performed, and initial biochemical analysis suggested aseptic meningitis. He was started on ceftriaxone and acyclovir according to protocol. Viral panel analysis of cerebrospinal fluid (CSF) was negative. Table 2 summarizes the CSF analysis. 

**Table 2:** CSF analysis. CSF: cerebrospinal fluid; WBC: white blood cells.

CSF	Result	Biological reference
Appearance	Clear	
Glucose	88 mg/dL	40-70 mg/dL
Protein	30.3 mg/dL	15-40 mg/dL
WBC	No cells	
Culture and sensitivity	No growth after 72 hours	
Meningoencephalitis panel	No organism detected	

**Figure 1:**
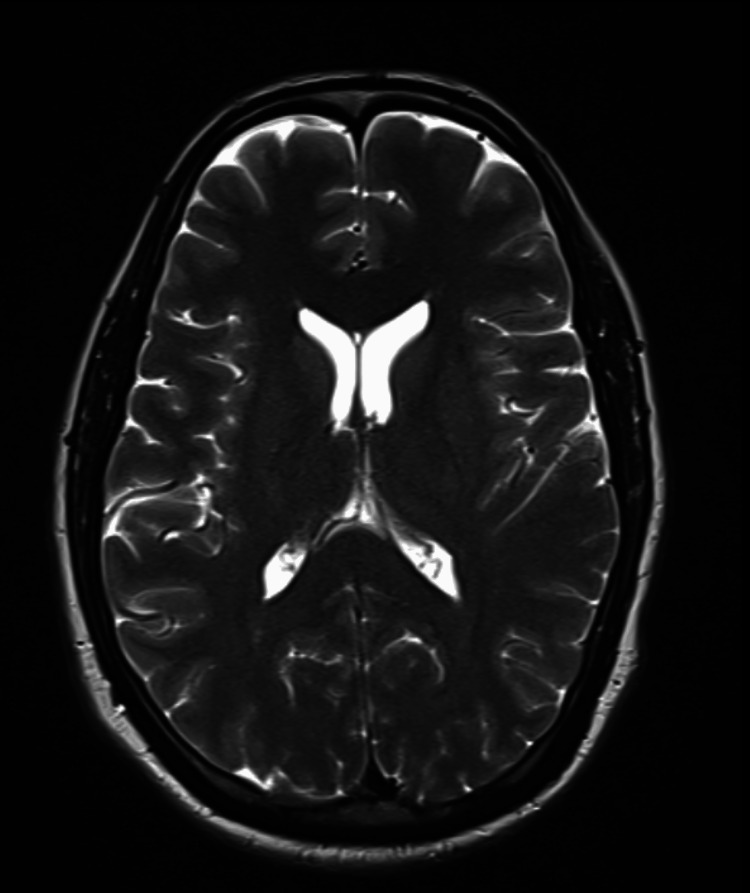
T2-weighted MRI of the brain parenchyma showing normal findings. MRI: magnetic resonance imaging.

After three days of conservative management with antibiotics, the patient's sensorium worsened. We repeated the biochemical parameters and found urea at 45 mg/dL, creatinine at 0.7 mg/dL, and electrolytes within normal limits. We performed a bone marrow aspiration for pancytopenia and found no abnormal cells. The ultrasound of the abdomen revealed a normal-sized kidney with maintained corticomedullary differentiation. A chest radiograph revealed a normal study. The antinuclear antibody test showed a positive result (4+) with a homogenous pattern. An antinuclear antibody profile was done to assess specific antibody levels, and the reports are presented in Table 3. We performed a magnetic resonance imaging (MRI) of the brain and found it to be normal (Figure 1).

**Table 3:** Antinuclear antibody profile. SS-A: Sjogren's syndrome-related antigen A; dsDNA: double-stranded deoxyribonucleic acid; DFS70: dense fine speckled 70 kDa.

Test	Result	Biological reference
Antibody to SS-A (Ro)	Positive + (20)	Negative
Antibody to dsDNA	Positive ++ (47)	Negative
Antibody to histones	Positive + (18)	Negative
Antibody to nucleosomes	Positive ++ (33)	Negative
Antibody to DFS70	Positive ++ (34)	Negative
Complement C3 level	<18.1 mg/dL	90-180 mg/dL
Complement C4 level	<7.07 mg/dL	10-40 mg/dL

With a diagnosis of SLE as a cause of aseptic meningitis, pulse steroid therapy (methylprednisolone 500 mg) was started, and the sensorium improved markedly, leading to a substantial overall recovery. He was continued on oral steroids (prednisolone 10 mg once daily). A renal biopsy was suggested because he had subnephrotic proteinuria against the background of SLE, but it could not be performed as he was not willing. He was not on regular follow-up after that.

**Figure 2:**
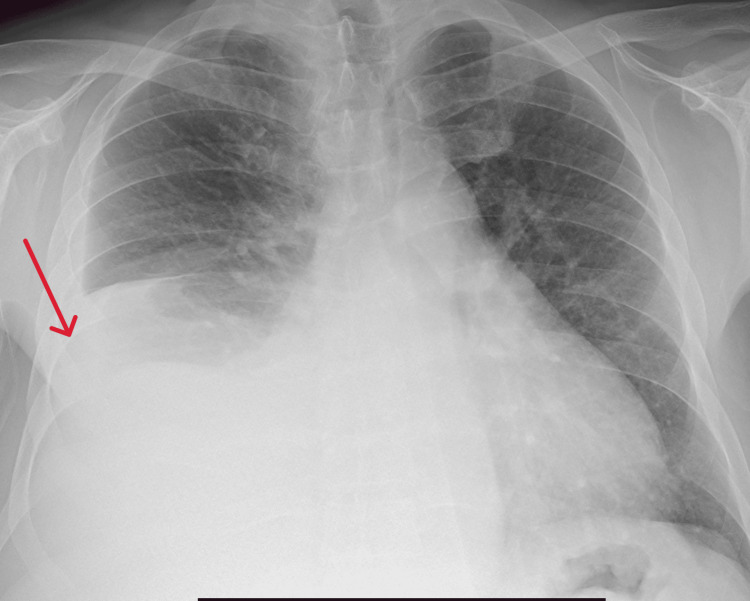
Chest X-ray showing right pleural effusion.

After one year, the patient presented with complaints of loss of weight, loss of appetite, and frothy urine. A 24-hour urine protein was 1180 g/day. An X-ray of the chest showed right pleural effusion (Figure 2). Diagnostic thoracentesis was performed and found to have exudative pleural effusion according to Light's criteria (pleural fluid lactate dehydrogenase (LDH) is greater than 2/3 of the upper limit of serum LDH). Table 4 summarizes the results. Because the patient was on long-term steroids, tubercular pleural effusion was ruled out. A diagnosis of serositis secondary to SLE was made.

**Table 4:** Pleural fluid analysis. LDH: lactate dehydrogenase; ADA: adenosine deaminase; MTB: *Mycobacterium tuberculosis*.

Test	Result	Biological reference
Pleural fluid		
Protein	4.6 g/dL	<3 g/dL
Glucose	68 mg/dL	80-140 mg/dL
LDH	1,180 U/L	<200 U/L
ADA	1.5 U/L	0-2.5 U/L
Albumin	1.8 g/dL	0.5-1.4 g/dL
MTB complex	Not detected	
Serum LDH	142 U/L	125-220 U/L
Serum protein	7.4 g/dL	6.6 -8.3 g/dL

**Figure 3:**
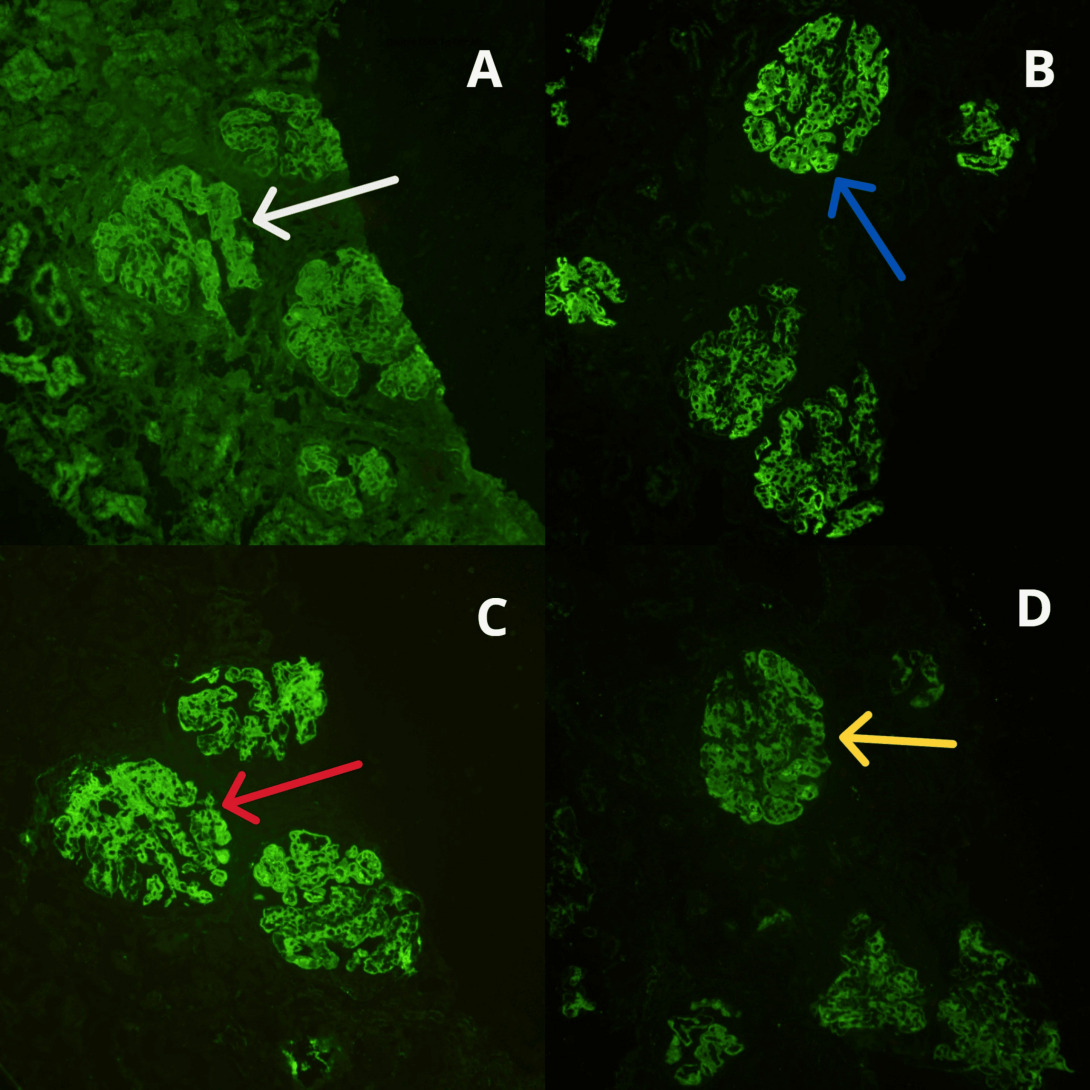
Under immunofluorescence staining, (A) granular positivity of C1q (white arrow), (B) granular positivity of C3 (blue arrow), (C) granular positivity of IgG (red arrow), and (D) granular positivity of IgM (yellow arrow) in glomerular capillary loops and mesangium. C1q: complement component 1q; C3: complement component 3; Ig: immunoglobulin.

**Figure 4:**
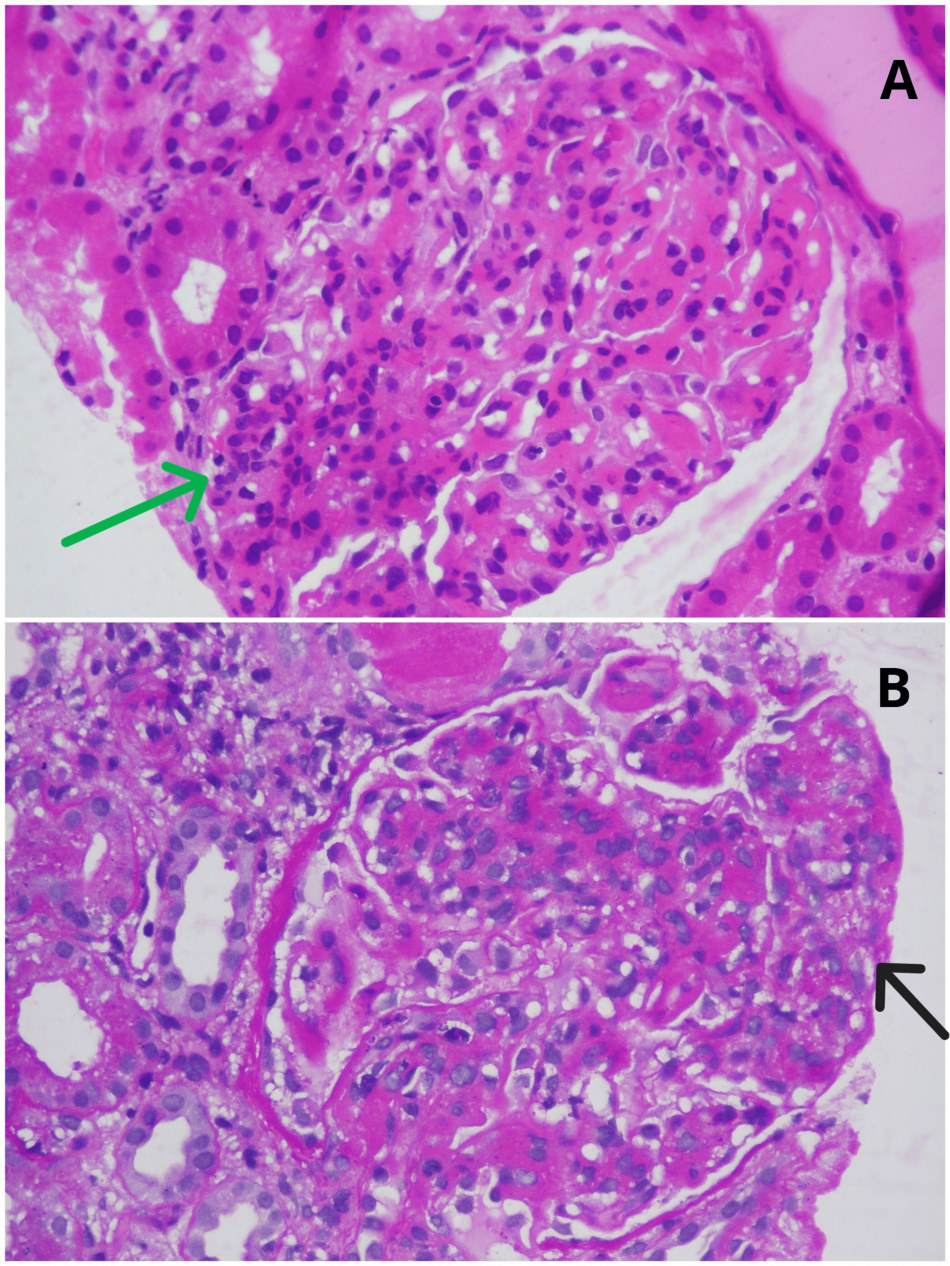
Under light microscopy, (A) hematoxylin and eosin staining shows endocapillary hypercellularity with neutrophilic infiltration (green arrow) and (B) PAS staining shows fibrocellular crescent formation (black arrow). PAS: periodic acid-Schiff.

A renal biopsy revealed characteristics consistent with class III lupus nephritis (focal proliferative lupus nephritis), as shown in Figures 3, 4. We initiated treatment with mycophenolate mofetil (MMF) and glucocorticoids according to treatment guidelines, resulting in no further episodes of relapse.

## Discussion

In 2019, the American College of Rheumatology (ACR)/European League Against Rheumatism (EULAR) laid down the diagnostic criteria for SLE. The hematologic, neuropsychiatric, mucocutaneous, serosal, musculoskeletal, immunologic, and constitutional domains of these criteria work together to diagnose SLE [4].

The medical literature reports that 4%-22% of SLE patients in published lupus series or populations are male [5]. The X-chromosome gene dose effect was proposed as a potential risk factor for pathogenesis, and there are epigenetic alterations such as methylation of DNA, innate immune system activation, and generation of autoimmunity [6,7]. Males with SLE tend to have similar symptoms as women, such as skin manifestations, arthritis, and central nervous system involvement. However, they experience less photosensitivity and more serositis, are diagnosed at an older age, and have a higher one-year mortality rate [8]. Kasitanon et al. found that SLE patients diagnosed after 50 years of age (p<0.001), male gender (p=0.005), and lower income (p<0.001) had a poor prognosis after adjusting other variables [9]. At the onset of SLE, male patients had higher rates of severe renal disease (p=0.064) and neuropsychiatric (NP) symptoms (p=0.055) compared to female patients [10].

After reviewing the available literature, we found that fewer than 1% of SLE patients had aseptic meningitis [11]. Most reported cases of meningitis in SLE have been chronic and may be drug-induced or infectious, such as tuberculosis. Immunosuppressants and non-steroidal anti-inflammatory drugs (NSAIDs) are potential causes of drug-induced aseptic meningitis. The literature has reported a few cases of aseptic meningitis presenting as the initial symptom of SLE in females [12]. This is one of the few cases in which aseptic meningitis is the first sign of SLE in a male patient. Overall, the stated prevalence of NP symptoms varies greatly, ranging from 14% to about 95% [13]. Borowoy et al. found statistically significant involvement of neuropsychiatric SLE in those who are antiphospholipid antibody positive [14].

Renal involvement in SLE has different forms, such as immune complex-mediated glomerulonephritis, tubulointerstitial disease, and vascular disease. Lupus nephritis typically begins within the first three years of the disease. Relapse could have been prevented if the patient had consented to a renal biopsy at the time of presentation.

The treatment aims to achieve remission while reducing organ damage. Hydroxychloroquine (HCQ), an antirheumatic drug that changes the course of the disease, treats mild lupus symptoms such as skin, joint, and mucosal involvement. We can use it alone or in conjunction with glucocorticoids; typically, low doses suffice. Induction therapy with immunosuppressive agents is necessary for SLE with significant organ involvement, followed by a maintenance dose to prevent relapse and flares.

## Conclusions

Although SLE is uncommon in males, its severity warrants physicians maintaining a low threshold for early investigation. Although there are some differences in how the disease manifests and its severity between sexes, the range of clinical and serological features is generally consistent. This case highlights how SLE can manifest with neurological symptoms like meningitis, potentially masking the underlying autoimmune cause if not promptly identified.
